# Reliability and Agreement of Intramuscular Coherence in Tibialis Anterior Muscle

**DOI:** 10.1371/journal.pone.0088428

**Published:** 2014-02-10

**Authors:** Edwin H. F. van Asseldonk, Sanne Floor Campfens, Stan J. F. Verwer, Michel J. A. M. van Putten, Dick F. Stegeman

**Affiliations:** 1 Department of Biomechanical Engineering, MIRA-Institute for Biomedical Technology and Technical Medicine, University of Twente, Enschede, The Netherlands; 2 Department of Clinical Neurophysiology, MIRA-Institute for Biomedical Technology and Technical Medicine, University of Twente, Enschede, The Netherlands; 3 Department of Neurology and Clinical Neurophysiology, Medisch Spectrum Twente, Enschede, The Netherlands; 4 Department of Neurology, Donders Institute for Brain, Cognition and Behavior, Radboud University Nijmegen Medical Center, Nijmegen, The Netherlands; 5 Research Institute MOVE, Faculty of Human Movement Sciences, VU University, Amsterdam, The Netherlands; University of Sydney, Australia

## Abstract

**Background:**

Neuroplasticity drives recovery of walking after a lesion of the descending tract. Intramuscular coherence analysis provides a way to quantify corticomotor drive during a functional task, like walking and changes in coherence serve as a marker for neuroplasticity. Although intramuscular coherence analysis is already applied and rapidly growing in interest, the reproducibility of variables derived from coherence is largely unknown. The purpose of this study was to determine the test-retest reliability and agreement of intramuscular coherence variables obtained during walking in healthy subjects.

**Methodology/Principal Findings:**

Ten healthy participants walked on a treadmill at a slow and normal speed in three sessions. Area of coherence and peak coherence were derived from the intramuscular coherence spectra calculated using rectified and non-rectified M. tibialis anterior Electromyography (EMG). Reliability, defined as the ability of a measurement to differentiate between subjects and established by the intra-class correlation coefficient, was on the limit of good for area of coherence and peak coherence when derived from rectified EMG during slow walking. Yet, the agreement, defined as the degree to which repeated measures are identical, was low as the measurement error was relatively large. The smallest change to exceed the measurement error between two repeated measures was 66% of the average value. For normal walking and/or other EMG-processing settings, not rectifying the EMG and/or high-pass filtering with a high cutoff frequency (100 Hz) the reliability was only moderate to poor and the agreement was considerably lower.

**Conclusions/significance:**

Only for specific conditions and EMG-processing settings, the derived coherence variables can be considered to be reliable measures. However, large changes (>66%) are needed to indicate a real difference. So, although intramuscular coherence is an easy to use and a sufficiently reliable tool to quantify intervention-induced neuroplasticity, the large effects needed to reveal a real change limit its practical use.

## Introduction

Recovery of walking after a lesion of the descending tract relies on neuroplasticity, reorganization of the function, structure and connections of the central nervous system in response to internal or external (i.e. training) stimuli [1]. Human walking requires integrated action of neural control circuitries at the spinal cord and brain [2]. Changes in these circuitries, reflecting neuroplasticity, can be obtained using coherence analysis of motor unit firing behaviour during walking.

Coherence derived from a pair of EMG signals, EMG-EMG coherence, quantifies the common oscillatory drive to a pair of muscles (intermuscular coherence) or to two parts of the same muscle (intramuscular coherence). EMG-EMG coherence in the beta (15–35 Hz) bands is considered to reflect the common corticospinal drive from the primary motor cortex to the muscles [3]–[8] whereas also spinal circuitries could potentially contribute [9], [10]. EMG-EMG coherence is an attractive approach to assess this common drive since it is easy to measure, requiring only the recording of EMG signals without the need to perturb or stimulate the system [2], [11]–[13].

EMG-EMG coherence can be applied during functional tasks like walking. Early studies explored the cortical involvement in the control of walking [3], [5], [7]. More clinically oriented studies showed that the EMG-EMG coherence in the beta band in patients with motor deficits resulting from stroke [14] or spinal cord injury [11], [12], [15] is decreased. These decreases indicate that beta-band EMG-EMG coherence depends largely on the integrity of the corticospinal tract. In spinal cord injury subjects, the decreased intramuscular coherence is related to impairments during walking [11]. Furthermore, quantification of EMG-EMG coherence has been used to monitor corticospinal drive as a result of a gait rehabilitation intervention. Norton and Gorassini [15] demonstrated that changes in locomotor function in spinal cord injury subjects after an extensive treadmill training program were accompanied by increases in corticospinal drive. This was considered to reflect neuroplasticity. These studies illustrate that EMG-EMG coherence can quantify the effects of interventions that aim to improve walking ability through promoting neuroplasticity like intensive (robot-aided) gait training or non-invasive brain stimulation [16].

Different variables are derived from the coherence spectra to capture changes in these spectra in a single value and assess effects on corticospinal drive. A commonly used derived variable is the area of coherence (Coh_area_) [11], [15], [17] which is the area under the coherence spectrum dwelling above the 95% confidence limit within a certain frequency band. Another typical variable is the peak coherence (Coh_Peak_) which is the peak value of the spectrum within a certain frequency band [7], [18].

While EMG acquisition is relatively easy, the processing of the signals for the calculation of coherence includes several choices. These may include (high-pass) filtering and rectification. Most experimental studies reporting EMG-EMG coherence used rectification of EMG signals before calculating the coherence. The need for this processing step is debated in recent studies [19]–[26]. Rectification has been suggested to enhance information about motor unit firing rate [1], [22], [24]–[27]. However, recent studies have stressed that rectification is a non-linear operation which has an inconsistent effect on power spectra and may obscure the detection of a common oscillatory drive to the muscle(s) [20], [21], [23]. The effect of high-pass filtering on EMG-EMG coherence has only recently attracted attention. High-pass filtering improves the “information density of the signals” as the filtered signals allow for better force estimation [28], [29]. Recently, Boonstra and Breakspear [26] were the first to explore the effect of high-pass filtering on EMG-EMG coherence. They showed that high-pass filtering with cutoff frequencies between 100–300 Hz increased the coherence.

To be able to use coherence variables to assess the effect of interventions, the reproducibility of these variables for repeated measurements should be assessed including the processing settings that result in the best reproducibility. In the current study we focus on intramuscular coherence. To our knowledge, there has not been an extensive study on the reproducibility of coherence variables. Reproducibility is the degree to which repeated measurements in stable study objects provide similar results and can be split up in reliability and agreement. Reliability assesses how well subjects can be distinguished from each other and is quantified by the intra-class correlation coefficient. Agreement is the degree to which repeated measures are identical and is quantified by the standard error of measurement and the smallest real difference [30], [31]. In order for a measure to be well suited for application in intervention studies, especially the agreement should be large, indicating that small effects can be shown [30]. Here we assessed the test-retest reliability and agreement of coherence variables calculated from muscular activity measured during treadmill walking in healthy subjects.

As several factors can influence the coherence spectra, we determined the reliability and agreement of the area of coherence and peak coherence for different conditions and processing settings. First, we determined the effect of walking speed. In more fundamental studies addressing cortical involvement during normal walking, healthy subjects generally walk at their preferred walking speed. Stroke survivors and spinal cord injury subjects often have a lower preferred walking speed. Therefore, we assessed the reliability during normal walking and slow walking. Second, as the discussion about the necessity of rectification is still unresolved, we will assess the reliability of coherence variables derived both from rectified and non-rectified EMG. Third, we will determine the effect of high-pass filtering with high cutoff frequencies. Finally, we will investigate how the variability of the coherence variables depends on the number of segments used to calculate the coherence spectra.

## Methods

### Subjects

Ten healthy volunteers (nine male; age range 18–25 years) with no history of neurological conditions participated in this study. Nine subjects were right leg dominant. Leg dominancy was determined using a combination of three tasks (leading leg when stepping up a platform, leg used to step out when pushed from behind, preferred leg to kick a ball) [32].

### Ethics statement

This study was approved by the Local Ethical Committee of the Medisch Specrum Twente, Enschede, the Netherlands. Subjects signed informed consent in accordance with the Declaration of Helsinki.

### Experimental design

The subjects participated in three experimental sessions separated by at least 1 week. These sessions were part of a double-blinded, crossover study to assess the effects of transcranial Direct Current Stimulation (tDCS). In each session subjects first performed baseline measurements before tDCS was applied. We use these baseline measurements to assess the test-retest reliability and agreement. Note that if tDCS would have any effects, they are relatively short lasting, less than 150 minutes. Carryover of effects to the baseline measurements of the subsequent session can therefore be excluded[33].

### Experimental procedures

In each of the sessions, subjects walked on the treadmill during baseline measurements for 3 minutes at 2.5 km/h and for 2 minutes at 5 km/h. These durations were chosen such that at least 100 complete gait cycles were obtained for each walking speed. Before recording started in each of the blocks, subjects were given time to get used to walking on the treadmill. The order of the walking speeds was randomized across subjects and sessions.

To obtain an indication of the variability of the coherence variables within one measurement session and to investigate the influence of the number of segments included in the coherence analysis on the coherence variables, three subjects (# 1, #5 and #10) participated in additional session(s). The coherence variables obtained from these subjects in the regular sessions were not at the extremes of the ranges obtained from the complete subject population in the regular sessions. Here, these subjects walked longer on the treadmill: 10 min blocks at 2.5 and at 5 km/h. Two of three subjects participated in one additional session, the third subject participated in two additional sessions. These were performed on separated days to allow investigation of the reproducibility of the coherence variables for longer trials as well.

### Recordings

Subjects walked on a split-belt instrumented treadmill (Y-mill, Forcelink, Culemborg, the Netherlands). EMG signals were recorded via disposable Ag-AgCl electrodes (1 cm^2^ recording area, type H93SG, Tyco Healthcare/Kendall, Mansfield, MA, USA) placed in a bipolar configuration over the right and left proximal and distal part of the M. tibialis anterior (TA) muscles. The two electrode pairs on the TA were separated by at least 10 cm to avoid cross-talk and detection of activity from the same or overlapping motor unit territories [12], [34]. Electrode locations were referenced to anatomical landmarks to enable duplication of placements for subsequent testing. One pair was applied just lateral and distal of the tuberositas tibiae and the other 1 cm from the distal end of the muscle belly. EMG Signals were sampled at 2048 Hz using compact measurement equipment (Porti, TMS International, Oldenzaal, The Netherlands) and sent to a computer for visual on-line display and later off-line analysis. Ground reaction forces underneath each foot were recorded with a sampling frequency of 1000 Hz using force sensors embedded in the treadmill.

### Data analysis

#### Intramuscular coherence analysis

Recorded signals were processed off-line using MATLAB 7.11 (the MathWorks Inc., Natick, MA, USA). EMG was processed using one of three combinations of filtering and rectification to investigate the effect of these processing steps on the coherence spectra and the derived variables. First, EMG was band-pass filtered at 10–500 Hz and left non-rectified (NR). Second, after band-pass filtering (10–500 Hz) the signals were rectified (HP10-R). Third, a high cutoff frequency of 100 Hz was used in the band-pass filter (100–500) and subsequently the data were rectified (HP100-R). All filters were fourth-order Butterworth filters with zero-lag.

The subsequent analysis (data segmentation and frequency analysis) was the same regardless of the previous EMG processing done. We used the measured vertical ground reaction forces below each belt to detect heel strike and toe off for every single step. These gait events were used to segment the EMG activity. TA is active during the complete swing phase, which lasts from approximately 60%–100% of the gait cycle (where 0 and 100% indicate heel strike of the concerned leg). As the timing of this muscle's burst is similar across the walking speeds used in this study [35], we selected for every step the segment between 60% and 100% of the gait cycle. Although the TA activity extends into the stance phase, we did not use this data to exclude any possible heel strike artifacts from the analysis [7]. From each regular walking session, 100 segments were used to calculate the coherence.

Each segment was multiplied with a Hann window and padded with zeros to a length of 2048 samples (1 s), resulting in a frequency resolution of 1 Hz. All segments were transformed to the frequency domain using the fast Fourier transform. The power spectral density (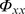) and cross spectral density (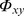)were calculated as
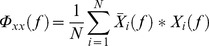
(1)and
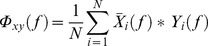
(2)where *X_i_(f)* and *Y_i_(f)* are the Fourier coefficients at frequency *f* estimated from the i^th^ data segment of the proximal TA and distal TA respectively, N is the total number of segments and the bar indicates the complex conjugate.

The (magnitude squared) coherence, *Coh_xy_* was calculated between signals using
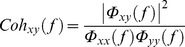
(3)


Intrauscular coherence was calculated using the power spectra from and the cross spectra between the proximal TA and the distal TA. Coherence is a spectral measure between 0 and 1 for the linear association between two signals. Zero indicates no linear relation, one indicates perfect, noise free, linear relation between the signals at that frequency [36]. The analysis steps above (zero-padding, calculation of spectral densities and coherence spectra) were performed using the Fieldtrip Toolbox for Matlab [37].

Intramuscular coherence is defined to be significantly larger than zero at a certain frequency when it exceeded a confidence limit (*CL*) with a probability of 95% (α  = 0.05). We determined the CL as 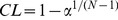
(4)where α is the desired significance level [38].

In addition, the cumulant density function, i.e. inverse Fourier transform of the coherence spectrum, was calculated as a time domain measure of association between EMG signals.

We calculated the coherence spectra and cumulant density function for every subject, for every session, for every speed for the dominant leg using each of the three differently processed EMG signals (NR, HP10-R and HP100-R). This resulted in a set of three coherence spectra per subject for every combination of walking speed and processing settings.

Similarity between these coherence spectra was quantified by calculating the correlation coefficient between all possible pairs of coherence spectra of these sets for the beta frequency band of 15–35 Hz. The individual correlation coefficients were averaged across subjects to obtain a single value for every combination of walking speed and processing settings.

#### Coherence variables

**Figure 1:**
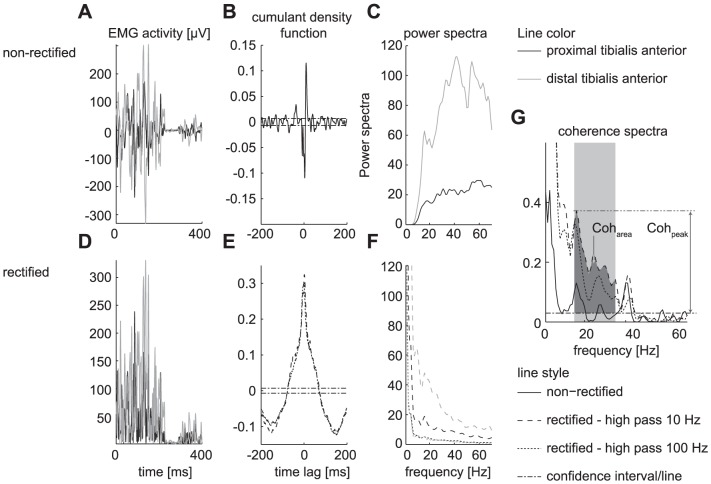
Rectifying and filtering of EMG signals has a large effect on power and coherence spectra. Representative data for a single subject (Subject #2) showing the effect of rectifying and filtering on the EMG activity (A,D), cumulant density function (B,E), power spectrum (C, F) and coherence spectrum(G).

We extracted two variables from the coherence spectra. First, the area of coherence (Coh_Area_) was defined as the area between the coherence spectrum and the CL in the beta frequency band (15–35 Hz) (see Fig. 1.). Second, the peak coherence (Coh_peak_) was defined as the maximum coherence within this frequency band and was expressed as the height above the CL (see Fig. 1.).

#### Reliability and agreement

We estimated the reliability by calculation of the intraclass correlation coefficient (ICC(2,1)) using a two-way random effects analysis of variance (ANOVA) [39]. This was done to separate the observed total variance of the variables into variance between subjects (MS_S_), variance between trials/sessions (MS_T_) and error variance (MS_E_). We calculated ICC(2,1) as
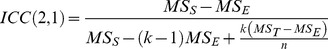
(5)where k is the number of sessions and n is the number of subjects. The ICC can range from 0 to 1, where ICC >0.75 is generally considered to indicate a good reliability, an ICC between 0.4 and 0.75 indicates moderate reliability and an ICC below 0.4 indicates poor reliability.

The agreement was estimated using two measures. First, we determined the Standard Error of Measurement (SEM). The SEM expresses how repeated measures of a subject on the same test tend to be distributed around the “true” value, assuming that there are no systematic errors. 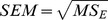
(6)


Second, we determined the Smallest Real Difference (SRD) [30], [40] also known as the Minimal Detectable Change (MDC). The SRD represents the smallest change necessary to exceed the measurement error of two repeated measures at a specified confidence interval (CI) [41] and was calculated for the 95% CI as:
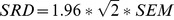
(7)where 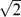 is used to account for the combined variance of two measurements.

The three variables, ICC, SEM and SRD were calculated using all subjects' individual Coh_Area_ and Coh_peak_ values for every combination of speed (slow, normal) and processing settings (NR, HP10-R and HP100-R) using IBM SPSS statistics 20.0. The SRD was expressed in absolute values as well as a percentage of the average variable value for that specific combination.

#### Within trial variation of coherence variables

We used the data from the extra sessions to investigate the within trial variation in coherence variables. The 10-min trials consisted of at least 400 segments. First we investigated how the coherence variables varied within these trials by calculating Coh_area_ and Coh_peak_ each consecutive subset of 100 segments. Second we investigate the effect of the number of segments used to calculate the coherence spectra and variables. The total number of segments was randomly divided into subsets consisting of 25, 50, 75, 100, 150, 200, 300 or 400 segments and the coherence spectra and variables were calculated for each of these subsets.

## Results

**Figure 2:**
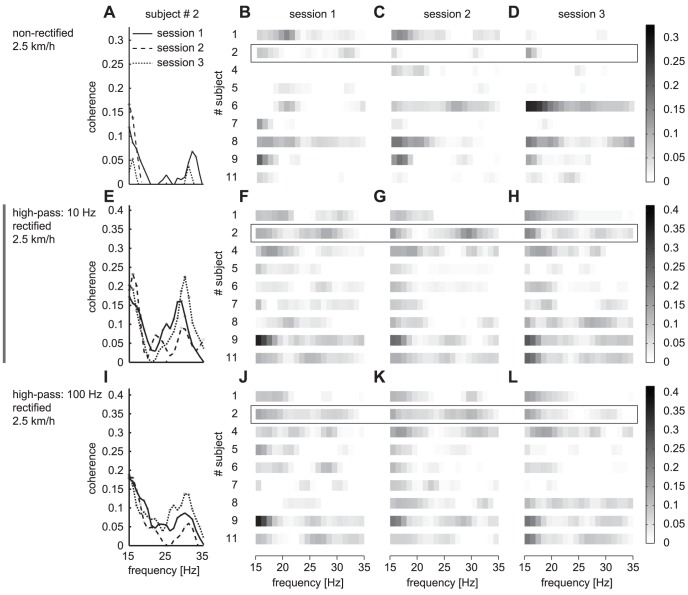
Coherence spectra are most consistent between sessions when estimated using rectified EMG signals. The most left graphs (A,E,I) depict the coherence spectra within the 15–35 Hz frequency range for the three different sessions for a subject #2. The other graphs show the presence and magnitude of the coherence for all subjects. Here the coherence magnitude is indicated in a gray scale, where a darker rectangle indicates a higher coherence. Each grayscale graph is a different combination of session and processing settings. For easy comparison with the other processing settings, HP10-R is depicted in the middle row. The confidence limit was subtracted from all coherence values.

All subjects showed significant Intramuscular coherence in all sessions for all speeds and for all processing procedures (Fig. 2). One subject was suspected to show cross talk between the two EMG signals (high intramuscular coherence, of about 0.3 over the complete frequency range of interest and a narrow peak in the cumulant density function of the rectified EMG [5]) and was left out of further analysis.

Unless specifically stated otherwise, the presented results are being obtained from rectified EMG (HP10-R). The results for the other settings will be discussed in relation to the HP10-R results.

### Coherence related measures determined from rectified signals show moderate reliability

During slow walking most subjects showed significant intramuscular coherence for more frequency bins within the 15–35 Hz range when rectified EMGs (HP10-R) were used for computation (see Fig. 2 E–H). Between sessions, the number of frequency bins, the location of these bins and the magnitude of coherence within these bins were quite consistent. This was reflected in marginally strong cross correlations between a subject's coherence spectra from different sessions, 0.56±0.23 (see Table 1).

**Table 1:** The across subject average correlations (µ ± std) between coherence spectra from different sessions.

Speed (km/h)	Proc.	Correlation
2.5	NR	0.30±0.38
	HP10-R	0.56±0.23
	HP100-R	0.48±0.30
5.0	NR	0.02±0.14
	HP10-R	0.48±0.17
	HP100-R	0.55±0.21

**Figure 3:**
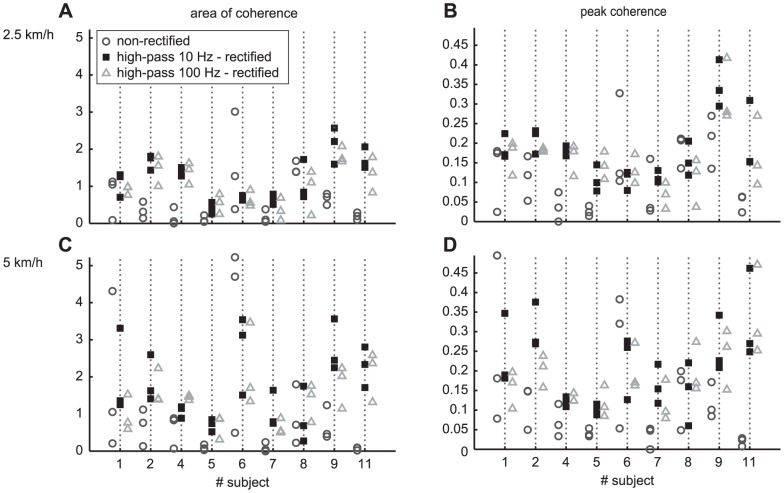
Coherence variables show considerable within-subject variability for HP10-R and even larger for different processing settings. Effect of processing settings on within and between subject variation in the area of coherence (A,C) and peak coherence (B,D) for slow (A,B) and normal (C,D) walking speeds. Each marker indicates the variable value of a single session. The different marker styles and colors depict different processing steps.

There was considerable within-subject variability for both coherence variables (see filled black squares in Fig. 3). The ICC was at the border for good reliability for both measures: 0.76 for Coh_area_ and 0.72 for the Coh_peak_ (see Table 2). The SRD values amounted to approximately 66% of the average value. For normal walking the correlations between coherence spectra tended to be lower, 0.48±0.17 (see Table 1). Furthermore, the ICC was only fair (see Table 2) and the SRD was approximately as large as the average variable value.

**Table 2:** Reliability and agreement of the coherence-related measures and the coherence spectra.

Speed	Proc.	ICC (2,1)		SEM		SRD		% SRD	
km/h		Coh_area_	Coh_peak_	Coh_area_	Coh_peak_	Coh_area_	Coh_peak_	Coh_area_	Coh_peak_
2.5	NR	0.47	0.41	0.54	0.07	1.48	0.20	245	176
	HP10-R	0.76	0.72	0.30	0.04	0.83	0.12	69	66
	HP100-R	0.60	0.57	0.37	0.06	1.03	0.16	100	98
5.0	NR	0.33	0.28	1.26	0.11	3.49	0.29	372	252
	HP10-R	0.47	0.48	0.75	0.07	2.08	0.20	122	94
	HP100-R	0.38	0.48	0.61	0.06	1.68	0.18	118	94

Intra class correlation (ICC(2,1)), standard error of the mean (SEM) and smallest real difference (SRD) and the SRD expressed as percentage of the mean value (%SRD) are shown for the area of coherence (Coh_area_) and peak coherence (Coh_peak_) for two different walking speeds (2.5 and 5.0 km/h) and different processing settings: non-rectified (NR), high-pass filtered with a cutoff frequency of 10 Hz and rectified (HP10-R) and high-pass filtered with a cutoff frequency of 100 Hz and rectified (HP100-R).

### Coherence related measures determined from non-rectified signals show fair reliability

Using non-rectified signals for estimating the coherence resulted in clearly different power and coherence spectra compared to the spectra estimated from rectified signals (see Fig. 1). Not only the magnitude of the coherence changed, but also the number and location of the peaks (see Fig. 2A-D vs. 2E–H and Fig. 1). The coherence spectra from the non-rectified signals showed less similarity between sessions. This was reflected in smaller and weak correlations between subject's coherence spectra (see Table 1).

The smaller similarity was also reflected in more within-subject variability in Coh_area_ and Coh_peak_ and in the measures for reliability and agreement (see Fig. 3, Table 2). The ICC for both coherence variables was low (<0.44) for both speed conditions and the SRD were high (>176% of the across subject variable average)(see table 2).

### High-pass filtering with high cutoff frequency does not further improve the reliability

Although high-pass filtering with a 100 Hz cutoff frequency (HP100-R) reduces the power of the signals considerably (see Fig. 1), the magnitude of the coherence and its distribution across the different frequencies were quite similar to the coherence when filtering with a cutoff frequency of 10 Hz (HP10-R)(Fig 2I-L vs. Fig 2E–H). Still, the ICC for Coh_area_ and Coh_peak_ are lower (fair to moderate) and the SRD was about 94% of the average variable value (Table 2).

### Within-session variation in coherence variables is close to between-session variation

**Figure 4:**
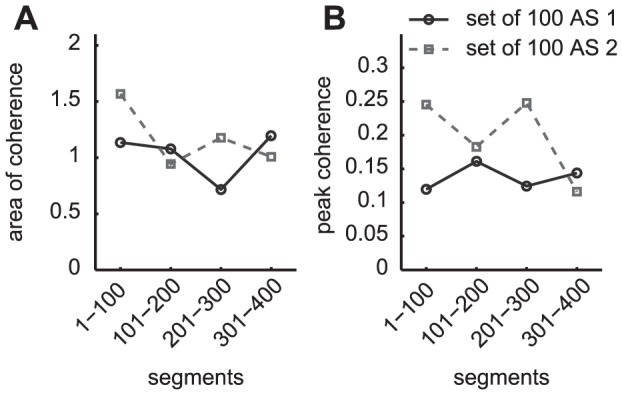
Coherence variables show within-session variation, which is about equal to observed between-session variation. Variation of area of coherence (A) and peak coherence (B) within a single 10-min walking trail at 2.5 km/h for subject #1 for the HP10-R processing. The values were calculated for consecutive subsets of 100 segments. The 10-min walking trail was performed in two additional sessions (AS) each indicated with a different line/marker style.

To better understand the origin of the variability of coherence variables, we evaluated the within-session variation of these variables in the additional 10 min-walking sessions performed by three subjects. Coh_area_ and Coh_peak_, as calculated from consecutive subsets of 100 segments show considerable variability within a single session (see Fig. 4). The observed variation was about equal in magnitude as the observed between-session variation for this subject (#1) in the three regular sessions as shown in Fig. 3 A and B and between extra session 1 and 2 (see Fig. 4).

This was a consistent finding for the slow as well as for the normal walking speed, for the other processing settings and for all three subjects that participated in these extra sessions (not depicted).

### Number of used segments influences the mean value of area of coherence but not peak coherence

**Figure 5:**
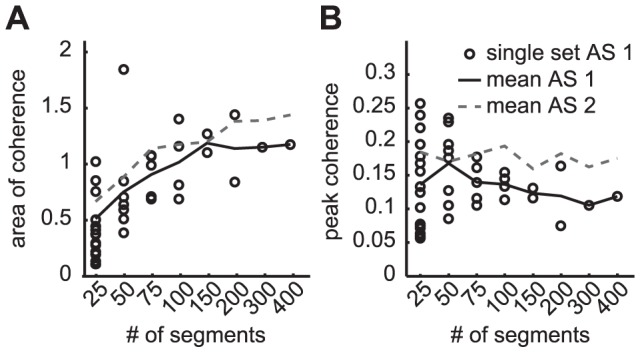
Area of coherence increases with number of segments used to calculate the cohernce spectrum. Influence of number of segments on the area of coherence (A) and peak coherence (B) for HP10-R. Data of subject #1 for the slow walking condition were used. Each marker indicates the value calculated from a subset containing the specified number of segments. These subsets were randomly taken from a total of 421 (additional session 1, AS1) or 418 (additional session 2, AS2) segments. The solid line indicates the mean value as a function of the number of segments for AS1. The dashed line shows the mean for AS2. The separate values for AS2 are not shown.

There was a large variability when few (25 or 50) segments were used to calculate the coherence variables. This variability tended to decrease with an increasing number of segments. Yet, a fair comparison is hampered as the number of available subsets also decreases with the number of segments. Strikingly, even when using 200 segments, the within-session difference between calculated Coh_area_ values can be as large as about 50% of the mean value (Fig. 5).

To determine the effect of the number of segments on the mean value of the coherence variables we calculated the mean for each number of segments. The mean Coh_area_ increased with the number of segments for all processing conditions, whereas Coh_peak_ did not show a clear trend (see Fig. 5, black solid lines). Similar trends for the variability and mean value were observed in the other subjects, for normal walking and also for the other processing settings.

The observed between-(extra)session variability is in line with the variability in the regular sessions. Using a larger number of segments to calculate the coherence values did not seem to influence the between-session variation. When using 400 segments the Coh_area_ differed about 22% in the second session compared to the first session (see Fig. 5A bold vs. dashed line) and the Coh_peak_ differed 46% (see Fig. 5B). For other processing settings and walking speed the differences were as large or larger, for non-rectified signals the increase in Coh_area_ was as high as 70%.

## Discussion

In this study, we quantified the test-retest reliability and agreement of variables derived from intramuscular coherence during walking. Intramuscular coherence is a measure of the common input to different parts of the same muscle and is applied as a measure of the corticospinal drive [4], [6], [8]. We found that the reliability and agreement of intramuscular coherence variables depends on signal processing settings (EMG high-pass filtering and rectification) and experimental condition (walking speed). Reliability and agreement were best for slow walking and using rectified signals that were high-pass filtered with 10 Hz. For this combination the reliability was on the limit of good for Coh_area_ (ICC = 0.76) and Coh_peak_(ICC = 0.72), but the agreement was still low given the smallest real difference of ±66% of the average variable values, indicating that a difference between two measurements should at least be this 66% to be considered as a real difference. For other conditions and/or signal processing settings the reliability was fair to poor and the smallest real difference was larger than 94%. Finally, we demonstrated that the intramuscular coherence values were influenced by the number of segments included in the analysis and can show considerable within-subject variation even within a single session.

There was no clear and consistent difference between the reliability and agreement of the variables derived from the coherence spectra: Coh_area_ and Coh_peak_. Both variables also show the same dependence on signal processing steps. Therefore, we cannot generally recommend using one variable over the other or specify in which conditions to use a certain variable.

Only one other study addressed the reproducibility of coherence variables measuring corticospinal drive [42]. However they used EEG-EMG coherence and quantified the peak value during isometric contractions of the hand muscles. They showed that there was no significant correlation between the peak values from different sessions. Despite the fact that the correlation they used only quantifies the strength of linear association between the two measures and does not provide direct information about reliability or agreement, the lack of correlation does indicate that the reproducibility was poor. So far, coherence related variables, being derived from EMG-EMG or EEG-EMG coherence, have shown poor reproducibility. Therefore, when these variables are used in intervention studies to assess neuroplasticity, their results should be interpreted with great caution. Still, the reproducibility of coherence variables has only been investigated for few muscles and tasks. The variables might be more reproducible for other combinations.

A limitation of this study is that we studied healthy individuals, and we do not know how the observations generalize to patients with motor deficits resulting from stroke or spinal cord injury. Different studies have shown that intramuscular coherence in these patients is lower than in healthy subjects [11], [12], [14]. Lower coherences, however, do not necessarily impact the reproducibility of coherence variables. Future reproducibility studies in these patients are therefore warranted.

The observed standard error of measurement was rather large for both intramuscular coherence variables, indicating that repeated measures of single subjects showed considerable variation around his/her “actual” score. These variations could at least partly be ascribed to errors in the measurement, but it might as well be that the underlying process is variable. This would imply that there is no “actual” score. It is well known that there are step-to-step fluctuations in gait and these fluctuations are not just a consequence of random noise in the system. In fact, Hausdorff and colleagues [43], [44] have shown that step-to-step fluctuations are related to fluctuations that occur hundreds of strides earlier. The neural circuits in the CNS responsible for these long-term fluctuations make the process time variant and as such variable. How much of the variation in the repeated measures can be ascribed to measurement error or variation in the underlying process is not known. The source of variability between measurements is not of that much importance for the reproducibility of the measure as all sources of variability negatively influence the reproducibility. However for the validity of coherence variables as a measure of corticospinal drive the source of variability is of importance. When the underlying process generating the common oscillatory drive to the muscles shows considerable variation, there is no “actual” intramuscular coherence and the validity of intramuscular coherence is affected.

Measures of EMG-EMG coherence can be vulnerable for cross-talk. Cross-talk compromises the validity of intramuscular coherence as a measure of common oscillatory drive to different motor unit territories. To prevent cross-talk the target muscle should be large, allowing large inter-electrode distances, and have restricted motor unit territories, which the TA muscle has. Hansen and colleagues [12] showed that significant intramuscular coherence was restricted to specific frequency bands (±12–32 Hz) and was small (<0.05) during isometric contractions when the electrode pairs were positioned 10 cm from each other, whereas with two pairs of electrodes closer together there was large significant coherence (>0.2) over a wide frequency range (0–500 Hz). The coherence spectra with 10 cm inter-electrode distances were similar to those obtained earlier from needle recordings of pairs of individual motor units in the same muscle [45], providing strong evidence that this electrode configuration indeed records activity from different motor unit territories. Furthermore, Roy and colleagues showed that the cat TA motor unit territories did not span the entire length of the muscle and had cross-sectional areas tapered along the proximodistal axis [34]. This makes it unlikely that electrodes located at both ends of the muscle belly will pick up activity of the same motor unit. Nevertheless, data recording and analysis needs to be done with great caution to prevent occurrence of cross-talk and/or detect it [11], [12]. All our subjects but one, who was excluded, showed significant coherence only in specific frequency bands of the spectrum and had a small and relatively broad central peak in the cumulant density function in all three sessions. Therefore, it is unlikely that cross-talk contributed to their coherence spectra and has influenced the reproducibility of the intramuscular coherence variables.

### Effect of processing settings and walking condition

The area of coherence showed a clear and gradual increase with the number of segments used to calculate the intramuscular coherence spectrum (Fig. 5A). Therefore the number of segments should be held constant between different measurements to make a fair comparison possible. Care should be taken when comparing the area of coherence from studies or conditions with different amounts of segments available and/or used in the analysis. Especially when the area of coherence is used to assess the effect of rehabilitation interventions, there is a considerable chance that individuals are able to walk for a longer time post compared to pre intervention [15]. The larger number of available segments for analysis would introduce a bias towards finding an increase in the area of coherence.

The reliability and agreements were determined from variables calculated using 100 segments. Possibly reliability and agreement would have been higher when more segments were used. This number was chosen, as our experience is that mildly affected stroke survivors or spinal cord injury subjects can walk 100 steps without signs of fatigue. The number of segments used in previous studies that investigated EMG-EMG coherence in neurological subjects varied from 70–72 [15] to 200–270 segments [14]. Our results from the additional sessions showed that also when using larger number of segments (200–400), there is still a considerable within-session and between-session variation in intramuscular coherence variables that is almost as large as the variation seen when using 100 segments. For instance, differences between Coh_area_ calculated from 2 sets of 200 segments (recorded in one trial) could amount to 50% of the average value. As a consequence, we do not expect large improvements in agreement and reliability when using more segments.

There is an ongoing debate on the question which EMG processing steps, i.e. rectification and filtering, are necessary in EEG-EMG and EMG-EMG coherence analysis [19]–[27]. Whether EMG rectification aids or impairs the detection of common drive and whether it can be detected at all, depends on many factors including the nature of the common drive (e.g. amplitude modulation or frequency modulation) [21], [26], the number of active motor units or force production [19], [27], [46] and the amount of common drive that motor units receive [21]. The complex interplay between all these factors cannot be controlled in experimental conditions and it is therefore difficult to determine which EMG processing setting results in the most valid quantification of corticospinal drive. However, a recent study of Ward and colleagues [27] provides strong evidence that rectification is a necessary preprocessing step to estimate coherence values. By using simultaneous recording of paired single motor units and surface EMG they demonstrated that rectified EMG could account for a larger part of the motor unit synchronization due to common oscillatory drive than non-rectified EMG. Our results are also in favor of rectification. We observed that rectification resulted in a shift of the power spectrum to the lower frequencies and an increase in the coherence in the beta band as reflected in an increase of the coherence variables, which was in accordance with recent work [26]. This increase of the coherence variables was accompanied by an increase in their reliability and agreement.

High-pass filtering with 100 Hz had no consistent influence on the intramuscular coherence variables compared to high-pass filtering with 10 Hz. This was in contrast with results from Boonstra & Breakspear [26] who showed that high-pass filtering with cutoff frequencies >100 Hz increased intermuscular coherence between bilateral leg muscles during quiet stance. We also showed that high-pass filtering resulted in a decrease of the reliability and agreement of the coherence variables. This shows that effects on reliability and agreement are not always in line with the effects on the magnitude of the coherence variables.

Previous studies did not show a clear effect of walking speed on EMG-EMG [12], [35] or EEG-EMG [47] coherence. In accordance with these studies we also observed no influence of walking speed on the coherence variables. The reliability and agreement of the variables was higher when subjects walked at slow speed, irrespective of the performed processing, showing again that the reproducibility does not necessarily depend on the magnitude of the coherence variables.

Most EMG-EMG and EEG-EMG lower limb coherence studies estimate the coherence in a dynamic situation like walking and only few during static contractions. Assessing coherence during dynamic movements is attractive as it quantifies (recovery of) the cortical involvement in that movement. However, as indicated earlier, there is considerable variability in the execution of these movements, which is detrimental for the coherence. Therefore, a better-controlled task like isometric force production might be more appropriate. Only one study quantified EMG-EMG coherence during walking and isometric force production [15] but the reported data do not allow a quantitative comparison. For EEG-EMG coherence, Gwin & Ferris [48] recently showed that the maximal coherence in the beta band did not significantly differ between a dynamic isotonic contraction and a static isometric contraction. Still, as stated earlier, from the (lack of) effects on the magnitude of the coherence variables, the effect on their reproducibility cannot be inferred. Future studies should address the reproducibility of EMG-EMG coherence variables in static conditions.

### Conclusion

Our study demonstrates the importance of determining the reproducibility of coherence variables. These variables are rapidly growing in interest as a measure of corticospinal drive to investigate the neuroplasticity of the CNS. The reproducibility of these variables is largely unknown. We focused on a subclass of coherence, being intramuscular coherence during walking. Our findings indicate that the reliability of intramuscular coherence variables obtained during walking was on the limit of good only under specific conditions and processing of EMG data (slow walking, using rectified signals). Still, their use in interventions studies is hampered by the low agreement. These results cannot be generalized to other muscles and tasks. Reproducibility should be separately assessed for other circumstances before being used in an intervention study.
